# Establishment of an acute arterial mesenteric ischaemia model in canines with an endovascular approach

**DOI:** 10.3389/fvets.2024.1373914

**Published:** 2024-06-13

**Authors:** Yadong Shi, Yangyi Zhou, Yuan Yuan, Jie Kong, Maofeng Gong, Liang Chen, Xu He, Haobo Su, Jianping Gu

**Affiliations:** Department of Vascular and Interventional Radiology, Nanjing First Hospital, Nanjing Medical University, Nanjing, China

**Keywords:** acute mesenteric ischemia, animal model, autologous thrombus, endovascular, thromboaspiration

## Abstract

**Purpose:**

This study aimed to evaluate the feasibility of establishing an arterial acute mesenteric ischemia (AMI) model in canines using transcatheter autologous thrombus administration.

**Materials and methods:**

Ten canines were divided into the experimental group (Group A, *n* = 5) and the sham group (Group B, *n* = 5). The canines in Group A received thrombus administration to the superior mesenteric artery (SMA) through a guiding catheter, while the canines in Group B received normal saline administration. Blood samples were collected and tested at baseline and 2 h after modelling. Canines in Group A underwent manual thromboaspiration after blood and intestine samples were collected. Ischaemic grades of intestinal mucosa were evaluated under light microscopes.

**Results:**

The AMI models were successfully conducted in all canines without procedure-related vessel injury or death. At the 2-h follow-up, the high-sensitivity C-reactive protein and D-dimer in Group A were significantly higher than in Group B (5.72 ± 1.8 mg/L vs. 2.82 ± 1.5 mg/L, *p* = 0.024; 2.25 ± 0.8 μg/mL vs. 0.27 ± 0.10 μg/mL, *p* = 0.005; respectively). The mean histopathologic intestinal ischaemic grade in Group A was significantly higher than in Group B (2.4 ± 0.5 vs. 0.8 ± 0.4, *p* < 0.001). After a median of 2 times of thromboaspiration, 80% (4/5) of the canines achieved complete SMA revascularisation.

**Conclusion:**

This experimental study demonstrated that establishing an arterial model in canines using endovascular approaches was feasible. The present model may play an important role in the investigation of endovascular techniques in the treatment of arterial AMI.

## Introduction

Acute mesenteric ischemia (AMI) is caused by the sudden interruption of blood supply that is insufficient to meet the metabolic demands of the visceral organs (1). Although AMI only accounts for 0.09–0.2% of all acute admissions to emergency (2), AMI is a life-threatening scenario with a mortality over 50% (3). More than half of the cases with AMI are attributed to acute embolisation of the superior mesenteric artery (SMA) (1, 2). The optimal treatment for embolic AMI remains under debate (4). Although various endovascular revascularisation (EVR) options, including local thrombosis, mechanical thrombectomy, angioplasty, and stent placement, have been introduced for the treatment of AMI, open revascularisation remains an important treatment for AMI (5). Compared with open revascularisation, EVR has shown superiorities in improved mortality, cost-effectiveness, decreased bowel resection, short bowel syndrome, and length of hospital stay (6–8). However, despite the increasing use of EVR techniques for embolic AMI in recent years (9), the high mortality of AMI has not significantly changed (9, 10). Moreover, owing to the absence of comparative studies, no endovascular treatment has proven superior over the other treatments (11). Mechanical thrombectomy for AMI may be associated with rapid revascularisation (12), whereas this technique has not been well validated and no dedicated thrombectomy devices for AMI are available. In order to find the optimal EVR techniques and reduce the mortality of AMI, a proper animal model may help to solve this dilemma.

The rodent model has the advantages of low cost and easy maintenance, and it is the most commonly used *in vivo* AMI model (13). However, the rodent model is not suitable for evaluating EVR techniques due to the relatively small vessel diameter (13). Moreover, traditional *in vivo* AMI models were performed by blocking SMA using a vascular clamp (14), surgical ligation (15), or coil embolisation (16). However, the models created by all these techniques are inappropriate for evaluating endovascular treatment for AMI. Therefore, it is urgent to establish an AMI model in a large animal with an appropriate vessel diameter that is suitable for evaluating the safety and efficacy of different EVR techniques.

In this study, we aimed to create a model of acute arterial mesenteric ischemia in canines using transcatheter autologous thrombus injection, mimicking the same condition in humans. Subsequently, percutaneous thromboaspiration was performed to further verify the feasibility of evaluating the EVR technique in the created canine model.

## Materials and methods

### Animals and preparation

Experiments were conducted following the Guide for the Care and Use of Laboratory Animals. The Institutional Animal Care and Use Committee approved all experimental procedures in this study (No. IACUC-2209011).

Ten female beagles (Changzhou Beile Experimental Animal Breeding Co., Ltd., license number: SCXK 2018-0007) weighing 15–20 kg were included for the modelling of arterial mesenteric ischemia. The 10 canines were divided into the experimental group (Group A, *n* = 5) and sham group (Group B, *n* = 5) randomly. Randomisation was performed using a computer random number generator. The canines in Group A received autologous thrombus administration using a guiding catheter, whereas the canines in Group B underwent normal saline administration. All canines fasted for 24 h without restriction to water before anaesthesia. Bowel preparation with polyethene glycol electrolytes (Beaufour Ipsen Industrie, France) was administrated via a gastric tube 1 day before modelling.

All canines were anaesthetised by using isoflurane (Shenzhen RWD Life Science, China) inhalation anaesthesia. Anaesthesia induction was achieved by 5% isoflurane with an oxygen volume of 5 L/min via mask inhalation. The anaesthesia was maintained using a 2.5–3% concentration of isoflurane with an oxygen volume of 2–3 L/min. After complete anaesthesia, canines were placed in an angiography suite in a fixed supine position. Cardiac and respiratory parameters were monitored throughout the procedures. The femoral artery and vein access were achieved using an 8 Fr and 4 Fr sheath (Radifocus Introducer, Terumo Corporation, Japan), respectively.

After completing all animal experiments, all canines were sacrificed with an intravenous lethal dose of potassium chloride.

### Thrombus preparation, animal modelling, and evaluation of thromboaspiration

**Figure 1:**
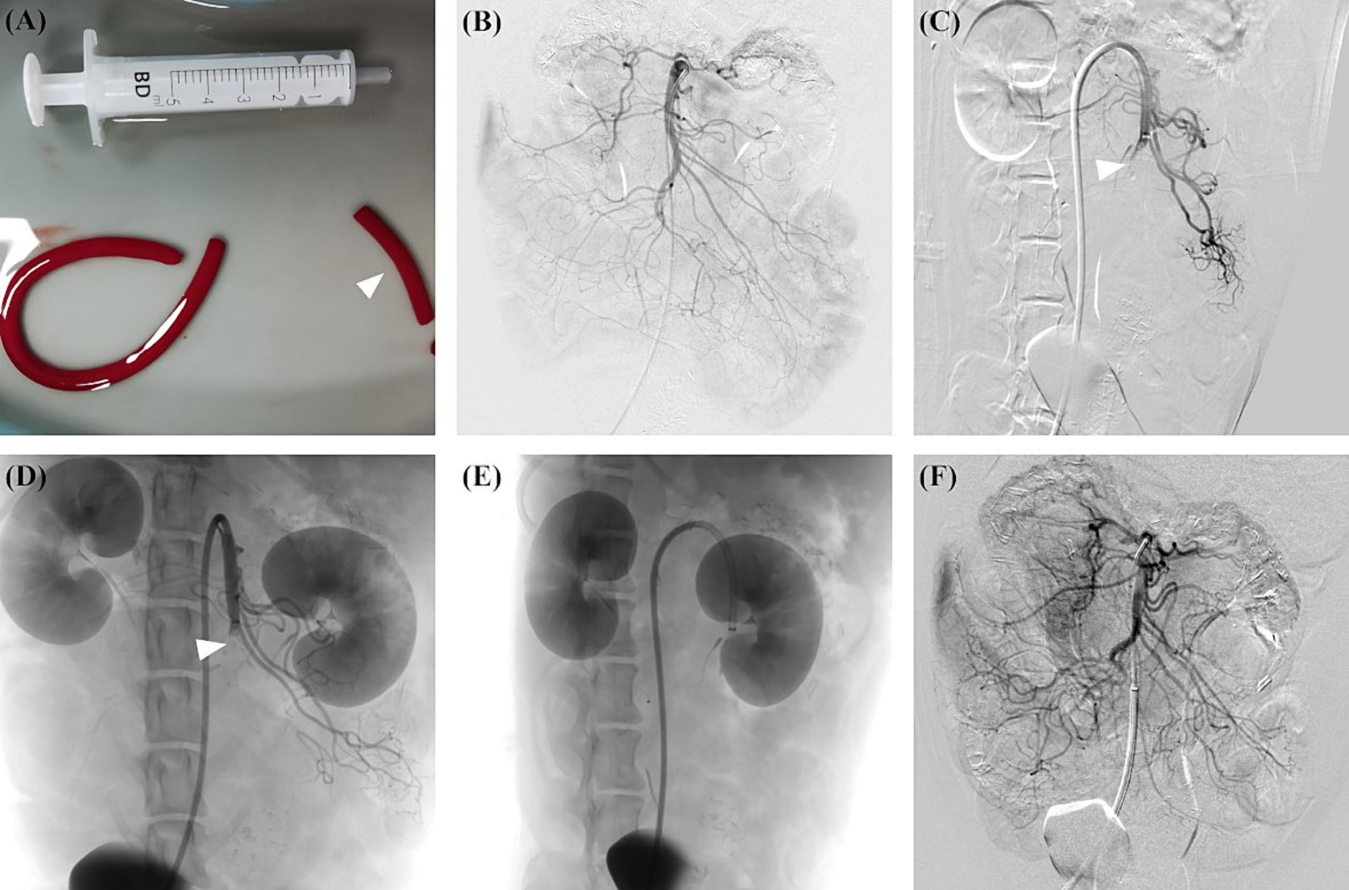
AMI model in canine was created using autologous thrombus administration with a guiding catheter, and subsequent thromboaspiration was performed. **(A)** An autologous thrombus was prepared and cut into 30 mm length pieces (arrowhead) before the modelling. **(B)** Preprocedural angiography showed the overview of SMA. **(C)** A guiding catheter was placed at the ostium of SMA, and the thrombus was subsequently administrated. Postprocedural angiography showed the complete occlusion of the middle SMA trunk (arrowhead). **(D)** Follow-up angiography at 2 h after modelling showed the SMA occlusion (arrowhead) without obvious thrombus dissolution or migration. **(E)** Before thromboaspiration, the guiding catheter was advanced as close to the thrombus as possible. **(F)** After thromboaspiration, post-procedure angiography showed complete SMA revascularisation without distal embolisation. AMI, acute mesenteric ischemia; SMA, superior mesenteric artery.

Autologous thrombus was created by mixing 8 mL of blood collected from the femoral artery, 64 mg of fibrinogen from bovine plasma (Sigma-Aldrich, St Louis, Missouri, USA), and 200 units of lyophilised thrombin (Hunan Yige Pharmaceutical Co. Ltd., China) in a 10 mL syringe for at least 3 min (17). The mixture was transferred to a 5 mm diameter silicone tube and incubated at room temperature for at least 60 min. The thrombus was then cut into approximately 30 mm length pieces before administration. Figure 1A shows the autologous thrombus prepared before administration.

The angiography of SMA was performed using a multipurpose angiographic catheter (Tempo, Cordis, USA). Visipaque (320 mg/mL, GE Health Care, USA) was used for angiography. The proximal and distal SMA diameters were measured. After the preprocedural angiography, an 8 Fr guiding catheter (Neuron MAX, Penumbra, USA) was placed in the proximal segment of SMA. For canines in the experimental group, the prepared thrombus was administrated through the guiding catheter with the assistance of a Y-shaped haemostasis valve (Access-9, Merit Medical, USA). The completion angiography was performed to assess the distribution of the thrombus. For canines in the sham group, 10 mL of normal saline was administrated through the guiding catheter instead of the thrombus. Follow-up angiography was performed 2 h after the completion of the modelling to assess the thrombus migration, thrombus dissolution, and vascular recanalisation. The proximal SMA was defined as the segment between the ostium and the origin of the inferior pancreaticoduodenal artery, and distal SMA was defined as the vascular bed downstream from the ileocolic artery (18).

The canines in Group A received percutaneous thromboaspiration after the blood and ischemic intestine samples were collected. Percutaneous manual thromboaspiration was performed as follows. A 0.035-inch hydrophilic guide wire was carefully navigated to pass through the occlusion segment. Then, an 8 Fr guiding catheter (Neuron MAX) was introduced through the guide wire and advanced as close to the thrombus as possible. After removing the guide wire, manual aspiration was performed with the assistance of a 60 mL syringe. Figures 1A–F illustrates the overall process of creating the arterial AMI model and performing subsequent manual thromboaspiration.

### Laboratory testing and histological analysis

Blood for laboratory testing was collected from the femoral vein. Blood samples were collected before animal modelling and 2 h after the modelling. High-sensitivity C-reactive protein (hs-CRP), white blood cell count (WBC), D-dimer, and lactate were assessed. CRP and WBC were tested by an automatic haematology analyser (BC-5390 CRP, Mindray, China). Lactate measure was made on a Vitros 4600 (Vitros, USA). D-dimer was measured by turbidimetric inhibition immunoassay incorporated in an Automated Blood Coagulation Analyser (SF-9200; Succeeder, China).

An exploration of the abdomen was performed to observe the location and severity of bowel ischemia before sacrifice. Tissue specimens were collected from the ischaemic intestine. The specimens were fixed in 10% formalin solution for at least 24 h and then embedded in paraffin wax, cut into 5-μm sections, and stained with haematoxylin–eosin (H&E). Three slices obtained from the same section were examined under light microscopy by a blinded, experienced pathologist. The intestinal wall was evaluated for histological examination and analysed for the microscopic pathological changes using the grading scale described by Chiu et al. (19) ranged from Grade 0 to Grade 5 as follows. Grade 0: Normal mucosal villi. Grade 1: Development of subepithelial Gruenhagen’s space, usually at the apex of the villus, often with capillary congestion. Grade 2: Extension of the subepithelial space with the moderate lifting of the epithelial layer from the lamina propria. Grade 3: Massive epithelial lifting down the sides of the villi. A few tips may be denuded. Grade 4: Denuded villi with lamina propria and dilated capillaries exposed. Increased cellularity of lamina propria may be noted. Grade 5: Digestion and disintegration of lamina propria; haemorrhage and ulceration.

### Statistical analysis

SPSS version 24.0 (IBM, Armonk, New York) was used for the statistical analysis. Count data are presented as *n* (%), and continuous data are presented as mean ± standard deviation. The Student’s *t*-test or Manne Whitney *U* test was used to compare the difference between quantitative data. The plots were generated with GraphPad Prism (v. 9.0; GraphPad Software Inc., USA). A *p* value <0.05 was considered statistically significant.

## Results

### Technical feasibility

The arterial AMI model was successfully achieved in all canines in Group A. No canines experienced any procedure-related complications, including haemorrhage, infection, arterial dissection, or perforation. All canines were included for analysis.

### Angiographic features and endovascular revascularisation

Preprocedural angiography revealed that the mean diameter of proximal and distal SMA was 5.1 ± 0.4 mm and 3.2 ± 0.4 mm, respectively. Angiography after thrombus administration showed that middle and distal SMA embolisation was noted in 3 (60%) and 2 (40%) canines, respectively. Branch vessel involvement was noted in 2 (40%) canines. The follow-up angiography 2 h after modelling revealed slight proximal thrombus dissolution in one canine, whereas thrombus migration or SMA recanalisation was not noted (Table 1).

**Table 1:** Post-modelling angiographic features of canines.

Parameters	Experimental group (*n* = 5)
Occlusion site	
Proximal SMA	0
Middle SMA	3 (60%)
Distal SMA	2 (40%)
Branch vessel involvement	2 (40%)
Follow-up angiography	
Thrombus dissolution	1 (20%)
Thrombus migration	0

Data are presented as *n* (%).

SMA, superior mesenteric artery.

After a median thromboaspiration of 2 times, 80% (4/5) of the canines in Group A achieved complete SMA revascularisation. Distal embolisation and vascular spasm related to thromboaspiration were noted in 2 (40%) and one (20%) canine, respectively. No other procedure-related complications were noted.

### Laboratory testing results and histopathology

**Figure 2:**
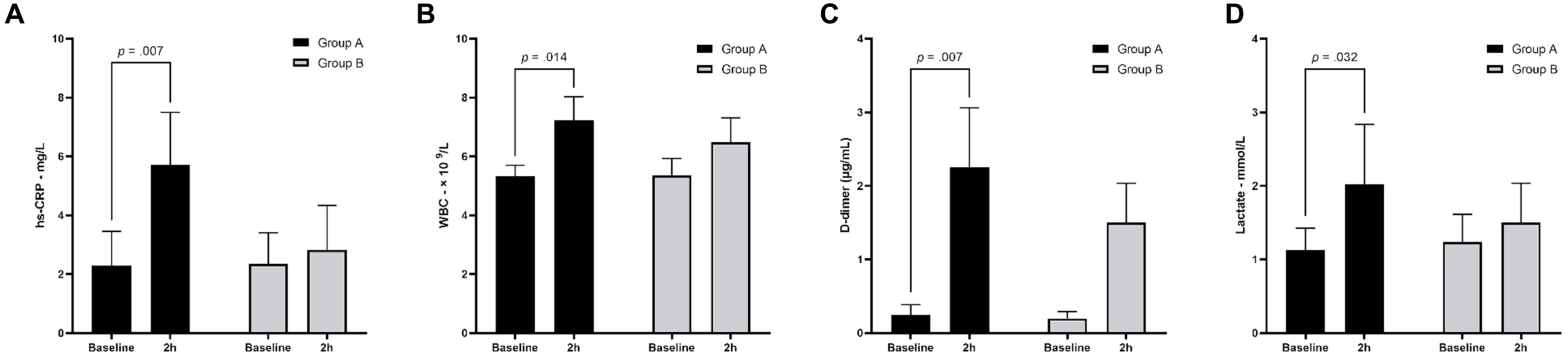
Laboratory testing results for canines at baseline and 2-h follow-up. At baseline, there is no significant difference between Group A and Group B. At 2-h follow-up, hs-CRP and D-dimer levels in Group A were significantly higher than in Group B. Compared with the baseline results, 2-h follow-up results of hs-CRP **(A)**, WBC **(B)**, D-dimer **(C)**, and lactate **(D)** were significantly increased in Group A. hs-CRP, high-sensitivity C-reactive protein; WBC, white blood cell count.

There is no significant difference in baseline hs-CRP (2.28 ± 1.2 mg/L vs. 2.34 ± 1.1 mg/L, *p* = 0.94), WBC (5.32 ± 0.4 × 10^9^/L vs. 5.32 ± 0.4 × 10^9^/L, *p* = 0.90), D-dimer (0.24 ± 0.14 μg/mL vs. 0.20 ± 0.10 μg/mL, *p* = 0.57), and lactate levels (1.12 ± 0.3 mmol/L vs. 1.24 ± 0.4 mmol/L, *p* = 0.61) in canines in Group A and Group B (Figures 2A–D). At the 2-h follow-up, hs-CRP and D-dimer levels in Group A were significantly higher than in Group B (5.72 ± 1.8 mg/L vs. 2.82 ± 1.5 mg/L, *p* = 0.024; 2.25 ± 0.8 μg/mL vs. 0.27 ± 0.10 μg/mL, *p* = 0.005; respectively). For canines in Group A, hs-CRP (5.71 ± 1.8 mg/L vs. 2.28 ± 1.2 mg/L, *p* = 0.007; Figure 2A), WBC (7.22 ± 0.8 × 10^9^ /L vs. 5.32 ± 0.4 × 10^9^/L, *p* = 0.014; Figure 2B), D-dimer (2.25 ± 0.8 μg/mL vs. 0.24 ± 0.14 μg/mL, *p* = 0.007; Figure 2C), and lactate levels (2.02 ± 0.8 mmol/L vs. 1.12 ± 0.3 mmol/L; Figure 2D) at the 2-h follow-up were significantly higher than at baseline.

**Figure 3:**
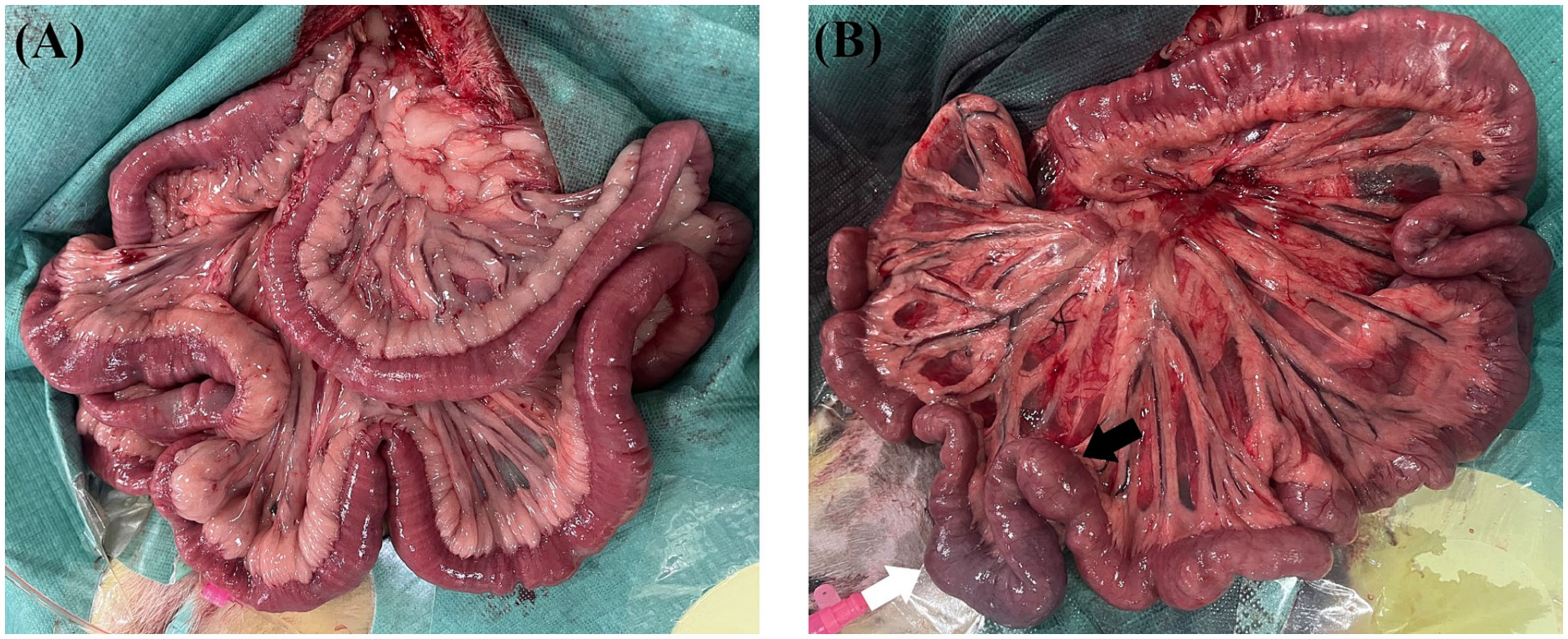
Intestine autopsy for canines in the sham group and experimental group. **(A)** No obvious intestinal ischemia was found in canines in the sham group after 2 h of modelling. **(B)** Intestine cyanosis (white arrow) and dilation (black arrow) were noted in canines in the experimental group after 2 h of modelling.

**Figure 4:**
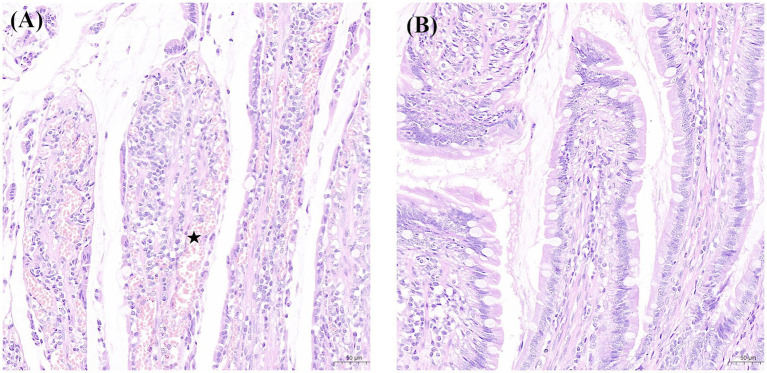
Findings of intestinal mucosa with H&E (×200) staining in the experimental and sham groups. **(A)** Denuded villi with lamina propria and dilated capillaries (pentagon) were noted in a canine in the experimental group. **(B)** Normal mucosal villi were presented in a canine in the sham group. H&E, haematoxylin and eosin.

**Figure 5:**
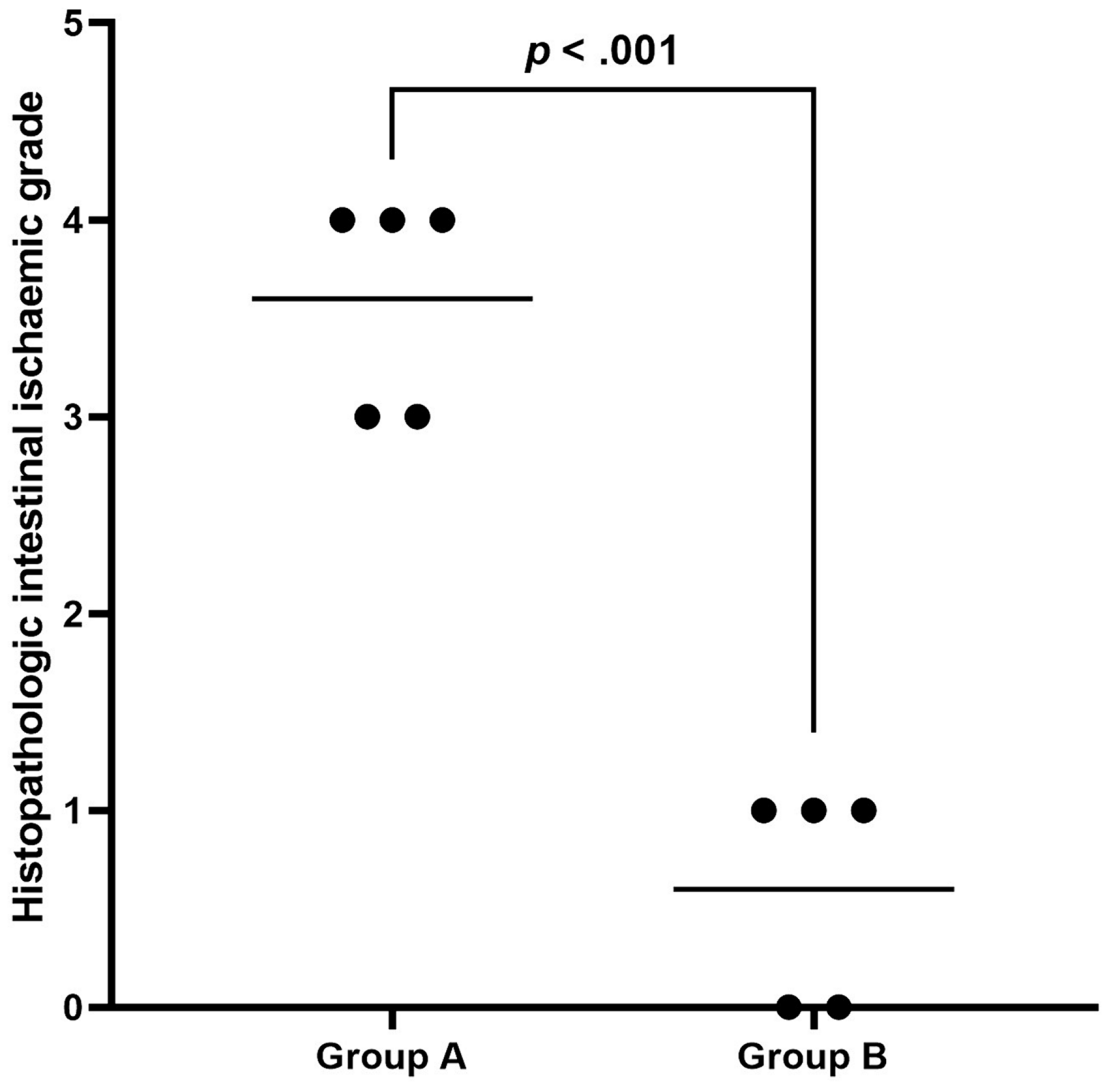
The plot shows the distribution of histopathologic intestinal ischaemic grade in Group A and Group B. The black line indicates the mean grade, and the ischaemic grade in Group A was significantly higher than in Group B.

During laparotomy, no obvious intestinal ischaemia was noted in the canines in Group B (Figure 3A), whereas intestinal cyanosis and dilation, which suggest intestine ischaemia, were observed in the canines in Group A (Figure 3B). No bowel perforation was observed in both groups. Figure 4 shows the pathological examination results of intestinal mucosa in canines in Group A (Figure 4A) and Group B (Figure 4B). The mean histopathological intestinal ischaemic grade in Gourp A was significantly higher than in Group B (2.4 ± 0.5 vs. 0.8 ± 0.4, *p* < 0.001) (Figure 5).

## Discussion

In the present study, we successfully established an arterial AMI model in canines using an endovascular approach by administrating autologous thrombus to the SMA. The present model was easy to operate and associated with a high success rate. The present model can be utilised for evaluating EVR techniques for AMI. To the best of our knowledge, this is the first report of an arterial AMI model created without the requirement for a vascular clamp, surgical ligation, or permanent embolic agent.

The treatment for AMI remains a huge challenge (20, 21). The successful management of AMI requires early diagnosis and prompt revascularisation (21). EVR seems to be a promising option compared to surgical revascularisation. Despite the development of endovascular techniques for the treatment of AMI, the mortality of AMI was not significantly reduced (9, 10). Although various endovascular techniques have been introduced, no device was designed dedicatedly for the treatment of AMI. Moreover, limited data comparing the different thrombectomy devices was available due to the rare incidence of AMI. Thus, the creation of an ideal animal model that mimics the pathophysiology of AMI and allows endovascular treatment is fundamental to early diagnosis and finding the optimal treatment.

The establishment of *in vivo* arterial AMI models has been reported in rodents and large animals (13–16). Rodent models were usually created by blocking SMA using a vascular clamp (14). These models were used to apply for research regarding imaging and biomarkers. The main disadvantage of rodent models is the small vessel size, which limits their applicability to fields such as endovascular research. Models created by clamping or ligation of the SMA or mesenteric vascular following laparotomy in large animals have been extensively reported (13, 15). The use of canines to recapitulate the human intestine has also been reported (13). Regarding the canine AMI model, surgical clamping of the SMA (22, 23) and mesenteric vascular (24, 25) were usually adopted. However, these models were created for evaluating intestinal ischaemia reperfusion injury and failed to simulate the clinical scenario of embolic AMI, which is usually caused by emboli. Another drawback of the surgical approach is that the damage of laparotomy may cause metabolic and haemodynamic changes. Models created by the endovascular approach were associated the minimal invasiveness and technical safety. Various embolic agents have been used in AMI models, including coils (16), polyvinyl alcohol particles (26), and n-butyl cyanoacrylate (27). Unfortunately, all the models mentioned above were also not suitable for evaluating EVR techniques. Autologous thrombus administration is an ideal method to mimic vessel occlusion, and it has been successfully used in acute ischaemic stroke models (28), whereas this method has not been reported in the AMI models. In the present study, we successfully established the AMI model in all canines in the experimental group, which demonstrated the feasibility of administrating autologous thrombus in SMA. Although no biomarkers had sufficient sensitivity and specificity to diagnose AMI (29), significantly increased hs-CRP, WBC, D-dimer, and lactate at 2-h follow-up might reflect the occurrence of AMI. Moreover, the histopathological intestinal ischemic grade in the experimental group was significantly higher than in the sham group. In summary, both blood biomarkers and intestinal histopathological results demonstrated that the present model well mimics the pathophysiological changes of AMI. Tual et al. reported that 28% of embolic AMI patients only had proximal SMA occlusion (18). However, no sole proximal SMA occlusion model was established in the current model. The mean diameter of the proximal and distal SMA was 5.1 ± 0.4 mm and 3.2 ± 0.4 mm, respectively. According to the diameter of SMA in the present study, administrating a thrombus through an 8 Fr catheter may result in thrombus migration with blood flow and lodging at the middle or distal stem of SMA. This is a potential limitation of the model established using the current approach. Transcatheter thrombus administration with the assistance of a dilated balloon catheter blocking the proximal segment of the SMA might be a feasible solution. However, future studies were required to demonstrate the feasibility.

There are several notable limitations to the present study. First, the present study only included a relatively small sample size. Future studies with large sample sizes are warranted to prove the feasibility and reproducibility. Second, most embolic AMI patients had atrial fibrillation (30), and the autologous thrombus created in the present study may not well mimic the organised embolus resulting from atrial fibrillation. Third, the present only evaluated the changes in 2 h after modelling. The evaluation of thrombus and intestine with an extended time was not available in this study. Fourth, models with sole proximal SMA occlusion were not available using the current endovascular approach. Based on our knowledge, this study is the first to report the feasibility of establishing an arterial AMI model through autologous thrombus administration in canines despite the limitations mentioned above.

In conclusion, this experimental study demonstrated that establishing an arterial model in canines using endovascular approaches was feasible. The present model well mimics the natural pathophysiology of arterial AMI in humans. Although the canine AMI model may increase costs when compared to the rodent model, given the fact that the optimal endovascular option for AMI has not been determined, the present model may play an important role in the investigation of various endovascular techniques in the treatment of embolic AMI. In addition, the present animal model may be helpful in developing dedicated thrombectomy devices for AMI.

## Data availability statement

The raw data supporting the conclusions of this article will be made available by the authors, without undue reservation.

## Ethics statement

The animal study was approved by Institutional Review Board of Nanjing First Hospital. The study was conducted in accordance with the local legislation and institutional requirements.

## Author contributions

YS: Conceptualization, Data curation, Formal analysis, Investigation, Methodology, Software, Validation, Visualization, Writing – original draft, Writing – review & editing. YZ: Data curation, Formal analysis, Investigation, Software, Validation, Writing – original draft, Writing – review & editing. YY: Data curation, Formal analysis, Investigation, Validation, Visualization, Writing – original draft, Writing – review & editing. JK: Formal analysis, Investigation, Methodology, Validation, Visualization, Writing – original draft, Writing – review & editing. MG: Data curation, Formal analysis, Investigation, Software, Validation, Writing – original draft, Writing – review & editing. LC: Conceptualization, Methodology, Project administration, Resources, Supervision, Validation, Writing – original draft, Writing – review & editing. XH: Conceptualization, Methodology, Supervision, Validation, Writing – original draft, Writing – review & editing. HS: Conceptualization, Methodology, Project administration, Supervision, Validation, Writing – original draft, Writing – review & editing. JG: Conceptualization, Methodology, Project administration, Resources, Supervision, Writing – original draft, Writing – review & editing.
